# Sexual Satisfaction and Mental Health in Prison Inmates

**DOI:** 10.3390/jcm8050705

**Published:** 2019-05-17

**Authors:** Rodrigo J. Carcedo, Daniel Perlman, Noelia Fernández-Rouco, Fernando Pérez, Diego Hervalejo

**Affiliations:** 1 Department of Developmental and Educational Psychology, University of Salamanca, Salamanca 37005, Spain; rcarcedo@usal.es (R.J.C.); ferp95@usal.es (F.P.); diegoherva@usal.es (D.H.); 2 Department of Human Development and Family Studies, University of North Carolina at Greensboro, Greensboro, NC 27402, USA; d_perlma@uncg.edu; 3 Department of Education, University of Cantabria, Santander 39005, Spain

**Keywords:** sexual satisfaction, sexual abstinence, partner status, mental health, prison inmates

## Abstract

The main goal of this study was to investigate the association between sexual satisfaction and mental health, and the combined effect of two previously found, statistically significant moderators: partner status and sexual abstinence. In-person interviews were conducted with 223 participants (49.327% males and 50.673% females). The effect of sexual satisfaction on mental health and the interactions of sexual satisfaction × partner status, sexual satisfaction × sexual abstinence, and sexual satisfaction × partner status × sexual abstinence were examined using simple moderation and moderated moderation tests after controlling for a set of sociodemographic, penitentiary, and interpersonal variables. Results revealed a direct relationship between sexual satisfaction and mental health only for the sexually abstinent group. Partner status was not significant as a moderator. It seems that the lack of sexual relationships is more powerful as a moderator than the lack of a romantic relationship. Additionally, the sexually abstinent group showed lower levels of sexual satisfaction in those with a partner outside or inside prison, and lower mental health independently of the current romantic status, than sexually active inmates. These findings point to the importance of sexual satisfaction to mental health in sexual situations of extreme disadvantage.

## 1. Introduction

More than 10 million people are living in jails and prisons worldwide [1], and considerably larger numbers of ex-prisoners are living in society [2]. A high prevalence of mental health problems is present in prison populations [3]. There is also increasing epidemiological evidence that prisoners are more likely to suffer from mental health problems than the average population [4,5,6,7].

In the most representative Spanish study that included 28.8% of the inmate populations in five different prisons, the lifetime prevalence rate of mental disorders was 84.4%. The prevalence of any mental disorder in the last month before the time of interview was 41.2% [8]. These results were confirmed more recently by a study with a smaller sample size (*n* = 184), obtained from three prototypical Spanish prisons [9]. A total percentage of 90.2% inmates had suffered a mental disorder during their lives. Also, 55.2% were suffering a mental disorder at the time. Finally, in this study, the inmate population was 5.3 times more likely to have a mental health problem than the general population.

These mental health problems are risk factors for a range of adverse outcomes in prison and on release including self-harm [10], suicide [11,12,13,14,15,16], and violence inside prison [17], and reoffending in released prisoners [2,18,19].

In sum, most prevalence studies have been conducted in developed countries and consistently show that a very high proportion of prisoners suffer from poor mental health [3,20]. Despite the high level of need, these disorders are frequently underdiagnosed and poorly treated [20]. In addition, a growing literature documents the detrimental consequences of incarceration for mental health [21,22,23,24]. For example, early scholars believed being imprisoned is associated with having higher rates of mental health disorders than inmates would have had if they had remained in the community [25]. Massoglia found evidence of persisting elevated mental health issues in previously incarcerated individuals [26]. Furthermore, incarceration is negatively associated with finances [27], family ties [28], and physical health [29] as well as a greater risk for sexual victimization [20].

All this makes the mental health status of current and former prison inmates an important public health issue [3]. Following the World Health Organization’s definition, this study will consider mental health as not merely the absence of illness but "a state of well-being in which every individual realizes his or her own potential, can cope with the normal stresses of life, can work productively and fruitfully, and is able to make a contribution to her or his community” [30]. Thus, this concept includes mental illness but also understands mental health as a positive dimension of well-being [31].

One of the possible causes of prison inmates being an at-risk population for poor mental health is that they encounter difficulties in having a satisfactory sex life [32,33,34,35]. Linville found that approximately 75 percent of a sample of 100 male inmates in a minimum-security prison reported emotional problems due to sexual deprivation [36]. As a result of the sexual deprivation inmates experience, they may seek relief in alternative, less satisfactory and/or riskier ways [37]. Different studies have demonstrated a high rate of masturbation [38,39,40], and the presence of consensual homosexual behavior as alternative forms of sexual behaviors [41,42]. Such behaviors are sometimes coercive [43,44,45], and can lead to the transmission of sexual diseases such as HIV [46]. Conjugal visitations have been suggested as one possible solution. Consistent with this view, states that permit conjugal visits have lower instances of reported rape and other sexual offenses in their prisons [47]. Nonetheless, the low frequency of visits, the lack of good conditions [48], and their being restricted to married or committed partners limits the efficacy of conjugal visits.

All these experiences are evaluated by prison inmates determining their level of sexual satisfaction. Sexual satisfaction has been defined as “an affective response arising from one’s subjective evaluation of the positive and negative dimensions associated with one’s sexual relationship” [49] (p. 258). It is regarded as a fundamental dimension of the quality of sexual activity. Research on sexual satisfaction in prison inmates has generally shown very low levels of sexual satisfaction except for those with a romantic partner inside the same prison and those who did not remain abstinent [48,50,51]. Taken altogether, sexual needs are not well satisfied in prison.

Arguably, sexual satisfaction can be considered an essential component of general well-being and mental health. Empirically, higher sexual satisfaction is associated higher mental health and lower depression [52,53,54]. The recognition of the need to be loved, appreciated and cared for, and of the desire for intimate relationships that provide emotional sustenance and empathy, have been considered important aspects for maintaining mental health in prisons [30].

### 1.1. The Sexual Satisfaction and Mental Health Relationship Moderated by Partner Status and Sexual Abstinence

Research on the relationship between sexual satisfaction and mental health in prison inmates is in a fledgling state. Researchers have largely overlooked the part sexual satisfaction can play in inmates’ mental health and well-being. Research involving these variables conducted with other populations is more extensive.

#### 1.1.1. Research Conducted Outside Prisons

The consequences of a satisfying sex life are important areas of research that are gaining increasing attention in the psychological and medical literature, suggesting that sexuality maintains its importance even in the context of serious health concerns [55]. In this way, higher sexual satisfaction is associated with low levels of sexual anxiety [56,57], low psychopathological symptoms [57,58], and good mental health [59,60].

Furthermore, fostering patients’ quality of life and mental health are key aims of health care in which subjective factors are commonly seen as central [61]. One subjective factor that has received very little attention is patients’ sexual satisfaction, although Mallis et al.’s results showed that sexual satisfaction and quality of life are “strongly connected” (p. 447) [62]. Other research has found sexual dissatisfaction is higher in patients with depression than in those without depressive symptoms [63].

Turning to relationship status and its role in the association between sexual satisfaction and mental health, in both non-clinical and clinical samples, partnered compared to single individuals have tended to report higher sexual satisfaction and sexual activity [52,64,65]. Having a partner does not necessarily mean that couples live together or that they have an active sex life, but it increases the likelihood that partners do have consistent sexual contact. Furthermore, tight-knit social structures such as being in a close relationship often, but not always, lead to better mental health outcomes [66]. Consistent with the beneficial view of tight structures, Holt-Lunstad, Birmingham, and Jones [67] and others (e.g., [68]) have found that being married is associated with better mental and physical health. Analyses such as this typically lump everyone together and do not examine other predictors of mental within subgroups.

Although the moderating effect of partner status between sexual satisfaction and mental health was not specifically investigated in the aforementioned studies of partner status, there is important evidence that the negative aspects of romantic life (e.g., loneliness and dissatisfaction, two aspects related to the fact of not having a partner or not having a satisfactory relationship for meeting one’s emotional needs) predict personal well-being more strongly than the positive aspects (e.g., marital satisfaction) [69]. Complementing the negative is stronger than the positive, other non-prison studies have found a strong relationship between sexual satisfaction and general well-being including mental health for those who had been sexually deprived due to the presence of sexual dysfunctions [70,71], physical disabilities [72], amputations [73], and having had germ-cell tumor therapy [74].

Other interpersonal variables, a category in which sexuality belongs [75], have a differential effect on mental health depending on partner status. For example, friendship quality only correlated significantly with depression among a group of college students without a romantic partner whereas no association was found in the group in a current romantic relationship [76].

Furthermore, Taleporos and McCabe compared the strength of this relationship for a group of people with and without sexual difficulty (physical disability vs. no physical disability) [72]. In this case, for both genders, the relationships between sexual satisfaction and indicators of mental health such as depression and self-esteem were stronger for people with physical disabilities than for able-bodied people. In other words, sexual satisfaction was a stronger predictor for the mental health of the group in a less favorable and more restrained condition. This situation might be comparable with the situation of sexually abstinent prison inmates who have shown much lower levels of sexual satisfaction than sexually active ones. In this comparison it is the sexually abstinent inmates who are in a more restrained and difficult situation.

Complementing Taleporos and McCabe’s results, Laumann et al. found that in the cluster of countries where average levels of sexual satisfaction were low (male-centered regimes; in a worse situation with a less freedom of choice) there was a stronger relationship between sexual well-being and happiness, which may be considered as an indirect indicator of positive mental health status, than in the cluster of countries where average levels of sexual satisfaction were higher (gender-equal sexual regime; in a better and free situation) [77]. If results in this vein generalize, one would then expect the association between sexual satisfaction and mental health to be stronger among sexually abstinent inmates. Also, based on prisoners’ previously mentioned negative feelings toward abstinence and the available data, we would expect the sexually abstinent inmates to have low sexual satisfaction.

#### 1.1.2. Research Conducted in Prison Contexts

Sexual satisfaction, mental health and other well-being-related measures have been found to be significantly correlated in studies conducted in prison settings [48,50,51,78]. The findings revealed that higher levels of sexual satisfaction were associated with higher levels of mental health and other well-being related measures.

Typically, in these studies the association between sexual satisfaction and mental health has been examined without considering the participants’ relationship status. The meaning of sexual experiences may vary depending on individuals’ romantic situation, especially among prison inmates who have stringent restrictions imposed on their sexual activities. In fact, research has shown that prison inmates without a partner or with a partner outside the prison had lower levels of sexual satisfaction and mental health than those inmates with a partner inside the same prison [48]. In a later study, a moderating effect of partner status on the relationship between sexual satisfaction and mental health was found. Lower sexual satisfaction was associated with lower mental health only for those without a partner [50]. These latter findings illustrate a pattern suggested in non-prison studies that the association between sexual satisfaction and mental health is intensified for those in a less desirable romantic status.

In arguing that a lack of sexual satisfaction can negatively impact prison inmates’ mental health, most authors [33,34,35,79] were referring mainly to inmates who had not had heterosexual relationships during their incarceration. Thus, these investigators were defacto ignoring inmates who were engaging in sanctioned sexual activities with their partners. Sexual satisfaction reflects a self-evaluation of one’s current sexual life; sexual abstinence refers to a complete lack of sexual relationships during a period of time. In reporting their sexual satisfaction, abstinent inmates were reporting on their satisfaction with not having sanctioned partnered sex whereas partnered inmates were reporting on the partnered sexual activities they were permitted to have. As has been found, an inmate may have been sexually abstinent during the last 6 months, yet show reasonable high sexual satisfaction [32]. By contrast, an individual may have been sexually active and show low sexual satisfaction. Thus in a noteworthy way, the referent for their judgments of sexual satisfaction is different for abstinent inmates than it is for partnered inmates.

This opens the possibility that the relationship between inmates’ sexual satisfaction and mental health may be different for sexual abstainers than for sexually active individuals. An earlier prison study found such a moderating effect [51]: sexual satisfaction was significantly associated with psychological health only for the group of inmates who had not had sexual relationships during the last 6 months, in other words, sexual abstainers.

In sum, previous research findings showed lower levels of sexual satisfaction and mental health in sexually abstinent inmates [32,51]. More importantly, an association between low sexual satisfaction and low mental health was only found for those who did not have a partner in the same prison (versus without a partner) [50] and those who remained sexual abstainers (versus non-abstainers) [32,51]. However, these two interaction effects have not been tested together to study (a) whether both are significant, (b) whether the proportion of variance for which they account is similar or different, and (c) whether there is a higher order interaction formed by sexual satisfaction, partner status, and sexual abstinence.

This study will focus on the new knowledge gained by including both abstinence and partner status. This current investigation also refines a previous study [50] because it includes three different partner statuses (no partner, partner outside of prison, and partner inside the same prison) instead of two (partner vs. no partner). Clearly there is a need of differentiating inmates with a partner inside or outside because these situations delineate different experiences.

In addition, this study benefits from a larger sample size and the addition of a set of control variables that have previously been demonstrated to have significant effects on mental health. Namely, poorer mental health has been exhibited by inmates who are younger, Caucasian [80], and married [80,81]; who have longer sentences and a longer expected time prior to their release [82]; who report poor general health [83]; who show higher levels of social and emotional loneliness [32,50,51,78]; and who, based on non-prison studies [84,85,86], masturbate more frequently. All these variables will be entered in the models as covariates.

#### 1.1.3. Research Questions

**Figure 1:**
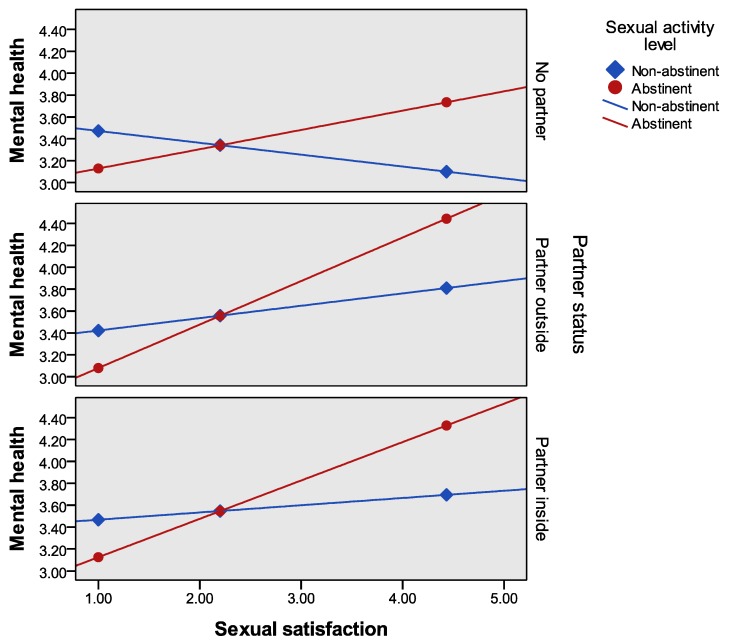
Sexual satisfaction × sexual activity level interaction associated with mental health for three different partner status groups.

Flowing from the summary of the aforementioned evidence found, two research questions emerge, a first and central question and an ancillary second one: (a) Will partner status and sexual activity level play a moderator role in the relationship between sexual satisfaction and mental health, after controlling for sociodemographic (sex, age, and nationality), penitentiary (total time in prison and estimated time to parole), and personal, social, and sexual well-being aspects (self-rated health, social, family, and romantic loneliness, and frequency of masturbation)? (see Figure 1) and (b) Will partner status and sexual abstinence be associated with inmates’ sexual satisfaction and mental health, after controlling for sociodemographic (sex, age, and nationality), penitentiary (total time in prison and estimated time to parole), and personal, social, and sexual well-being aspects (self-rated health; social, family, and romantic loneliness; and frequency of masturbation)?

## 2. Experimental Section

### 2.1. Participants

Participants for this study were entirely inmates from the medium-security Topas penitentiary, located in Salamanca (Spain). This prison houses men and women in the same prison but in different modules. The prison administration decided from which men’s and women’s modules the investigators could recruit participants. After stratifying by gender, 80% of the participants were randomly selected, whereas 20% were selected under a “snowball” sampling scheme [36]. Participants were excluded from this study if they (a) had been in prison for less than 6 months, the time considered necessary to become adapted to prison life and develop new relationships inside the facility; (b) did not speak Spanish or English; (c) had been diagnosed with a serious mental disorder; or (d) were not in an optimal condition to be interviewed (e.g., under the influence of drugs or expressing high levels of anxiety or distrust toward the interviewer). Only twelve potential participants declined being interviewed. All of the participants found the interview to be a positive experience.

Due to the difficulties collecting information from this specific population, we retained for analyses in the present report participants in two of Carcedo et al.’s previous studies [50,51] that had 119 and 173 participants, respectively. For this study, a sample of 223 inmates from 20 to 62 years old (M = 35.172, SD = 7.823) was used. We selected the increase in sample size to ensure reasonable power for testing the interaction effects of interest in the current analyses. This increase resulted in successfully having at least 10 participants per subgroup formed by crossing partner status (inside, outside, no partner) and sexual abstinence categories (abstinent vs. non-abstinent). Although males and females in prison are not equal in number, we selected a roughly equal number of male (*n* = 110) and female (*n* = 113) participants in order to explore the possible effect of sex on the results and, consequently, the results’ interpretation and discussion. Nationality was encoded in two levels: Spanish nationality (*n* = 103) and foreign, unspecified origin country (*n* = 120). Regarding the two moderators in this study, 76 inmates had no partner (34.080%), 61 had a partner outside the prison (27.354%), and 86 had a partner inside the prison (38.565%); also, 122 inmates reported having had sex in the last six months (54.709%) and 101 kept sexually abstinent (45.291%).

In comparison with inmates with a current romantic partner outside the prison, those in a relationship inside the same prison presented a higher frequency of in-person contact (*t* (145) = −12.413, *p* > 0.001; outside: M = 3.311, SD = 1.679; inside: M = 5.698, SD = 0.510; variable coding: 1 “never”, 2 “more than 6 months”, 3 “3–6 months”, 4 “each 1–2 months”, 5 “each 7–15 days”, and 6 “every day or almost every day”, and satisfaction with the current relationship (*t* (145) = −2.746, *p* < 0.001; outside: M = 3.510, SD = 1.678; inside: M = 4.358, SD = 1.326; variable coding: 1 “totally unsatisfied” and 5 “totally satisfied”) and lower duration of the union in months (*t* (145) = 3.588, *p* < 0.001; outside: M = 102.459, SD = 100.593; inside: M = 49.831, SD = 77.172).

All sexually active inmates reported they had engaged in heterosexual behavior at least once in the last six months. Regarding frequency, 63.934% of sexually active inmates had had sexual relationships at least once every 15 days, 24.590% every 2 months, and 11.475% every 6 months. Most of these sexual relationships occurred in conjugal visit rooms (76.471%), but also in other locations inside the prison (shared areas such as the sociocultural module, prison laundry, kitchen, gym, etc. (19.328%) and family visit rooms (1.681%)) and during furloughs outside the prison (2.521%). Inmates reported that their sexual relationships had included vaginal coitus at least once in the last 6 months. It is also important to mention that three sexually active participants also reported to have had some homosexual contact in prison. Finally, no sexually active inmate was convicted of sex crimes.

Preliminary analyses did not find any significant effect of sex, in the presence of partner status and sexual activity level, on sexual satisfaction and mental health nor a moderating effect between these two variables. Therefore, both sexes were analyzed together and sex was only included as a control variable in all the analyses.

### 2.2. Design and Procedure

This study used a short-term longitudinal design. Two interview sessions were carried out with a difference of a week between them. The main associated variable and control variables were extracted from the first interview and the outcome (mental health) was taken from the second one. Each participant was interviewed in a private room located in his or her prison module, separated from the rest of the inmates. The interviews were kept short (approximately 30 min without counting the time dedicated to create a good relationship) to ensure that participants did not get tired and to avoid “interrogation effects”.

All the interviews were conducted by the same interviewer to foster consistency. Before starting the interview, the interviewer spent a significant amount of time building a trustful relationship with every inmate (usually about 20–30 min, but depending on the speed of establishing rapport, in some cases it took up to 2 h). Afterwards, participants were invited to participate and were informed about the possibility of leaving the study whenever they wished to do so. Participants were informed about the confidentiality and anonymity of the study and all the participants signed consent forms. We consider that respecting all of these conditions is extremely important in collecting good-quality data from this population. Ignoring these conditions can easily increase distrust among the prison inmates. Finally, it is important to state that this study respected the norms of the Declaration of Helsinki’s ethical principles for medical research involving human subjects.

### 2.3. Measures

#### 2.3.1. Sexual Satisfaction

The sexual satisfaction subscale of the Multidimensional Sexual Self-Concept Questionnaire (MSSCQ) [87] was used to measure the main variable of this study. A total of five items were scored on a five-point Likert-type scale that ranged from 1 (not at all characteristic of me) to 5 (very characteristic of me). Cronbach’s alpha for this scale was 0.960.

#### 2.3.2. Moderating Variables: Sexual Activity Level and Partner Status

This variable was recorded as 0 for the inmates who had experienced sexual relationships in the past 6 months (non-abstinent), and 1 for the inmates who had not (abstinent). Sexual relationships were understood as any sexual behavior with another person including vaginal or anal intercourse, oral sex, and mutual masturbation and genital caresses, excluding kisses, hugs, and non-genital caress. Partner status was coded to have three categorical levels: no partner (0), partner outside (1), and partner inside the prison (2). Partner status was defined as a relationship deemed, in the inmate’s mind, as one that both partners considered serious.

#### 2.3.3. Outcome Variable: Mental Health

This construct was measured with the short Spanish version of the Psychological health subscale included in the World Health Quality of Life scale (WHOQOL-BREF) [88]. Six items were scored on a five-point Likert-type scale that ranged, with different labels, from 1 (not at all; very dissatisfied; never) to 5 (extremely-completely; very satisfied; always). Cronbach’s alpha was 0.709. Sample items include “To what extent do you feel your life to be meaningful?” and “How often do you have negative feelings such as blue mood, despair, anxiety, depression?” This scale was selected for multiple reasons: It is brief; it conceptualizes mental health not only as the absence of illness but also the presence of positive aspects of mental health; and its concurrent validity as indicated by its high correlation (*r* = 0.70) with the widely used SF-36 (36-Item Short-Form Health Survey) mental health subscale [89].

#### 2.3.4. Control Variables: Sociodemographic, Penitentiary, and Personal, Social, and Sexual Well-Being Variables

Considering sociodemographic variables, sex was codified as 0 for male and 1 for female inmates, age was asked directly to each inmate and confirmed against inmate penitentiary records for accuracy, and nationality was dichotomized into Spaniards (0) versus foreigners (1). Regarding penitentiary variables, total time in prison refers to the total time spent in prison for previous and current offenses. This information was collected by reviewing inmates’ penitentiary records, and it was recorded in months. Estimated time to parole was captured by asking the inmates how much time they expected to be in prison from that moment, based on the information they possessed. This variable was also computed in months.

With respect to personal, social, and sexual well-being variables, self-rated health was measured by asking the participants “in general, would you say your health is: excellent (4); very good (3); good (2); fair (1); or poor (0)?” [90]. The short version of the Social and Emotional Loneliness Scale for Adults (SELSA-S) [91] was used to measure both types of loneliness. SELSA-S consists of three subscales labeled (a) social loneliness, (b) family-emotional loneliness, and (c) romantic-emotional loneliness. Participants rated 15 items (five per scale) on a seven-point Likert-type scale that ranged from 1 (strongly disagree) to 7 (strongly agree). Cronbach’s alphas were 0.829, 0.898, and 0.840 for social, family-emotional, and romantic-emotional loneliness, respectively. Finally, masturbation frequency was codified into six levels based on the frequency inmates reported having masturbated during the last 6 months: (1) never, (2) less than once a month, (3) once or twice a month, (4) once or twice a week, (5) once a day, (6) twice a day or more.

Each scale or subscale score was obtained by adding the item scores and dividing them by the number of items answered. Higher scores represented higher levels in that dimension for all the variables included in this study.

### 2.4. Statistical Analysis

A 3 × 2, partner status (no partner, partner outside the prison, and partner inside) by sexual activity (abstinent vs. non-abstinent inmates) ANCOVA was used to first analyze the differences in sexual satisfaction and then performed again with mental health as the outcome variable. Each analysis controlled for sociodemographic, penitentiary, personal, social, and sexual well-being variables. If the partner status by sexual activity interaction between factors was statistically significant, Bonferroni post-hoc tests for multiple comparisons were conducted. Statistical significance was defined as *p* < 0.05.

The Breuch–Pagan test was conducted to test heteroscedasticity between sexual satisfaction and mental health. The macro heteroscedasticity test for SPSS [92] was utilized for this purpose. To study the relationships of sexual satisfaction with mental health and the moderating effects of partner status and sexual activity level, the PROCESS 3.2. macro for SPSS [93] was utilized. PROCESS’s models number one and two for two-way interactions (also called simple moderation), and three for the three-way interaction (also named moderated moderation) were used. Additionally, 95% confidence intervals were calculated based on 5000 bootstrap samples. The HC3 heteroscedasticity-consistent standard error estimator was applied [94] due to the violation of homoscedasticity. All the statistical analyses were conducted using the IBM SPSS 23 package (IBM Corp., Armonk, NY, USA).

## 3. Results

Descriptive information for the variables considered in this study are included in Table 1. With sexual satisfaction as the outcome variable, the 3 × 2 partner status by sexual activity level ANCOVA yielded significant effects for sexual activity level (F (1, 207) = 47.115, *p* < 0.001, η^2^_p_ = 0.185) and the partner status × sexual activity level interaction (F (2, 207) = 14.638, *p* < 0.001, η^2^_p_ = 0.124). Bonferroni post-hoc comparisons revealed lower levels of sexual satisfaction in sexually abstinent inmates in comparison with non-abstinent for those who had a partner outside (*p* < 0.001; abstinent: M = 0.838, SE = 0.237; non-abstinent: M = 2.979, SE = 0.178) or inside the same prison (*p* < 0.001; abstinent: M = 1.094, SE = 0.302; non-abstinent: M = 3.006, SE = 0.155). However, no differences in sexual satisfaction between abstinent and non-abstinent inmates were found for those who were not involved in a romantic relationship (*p* > 0.05; abstinent: M = 2.462, SE = 0.197; non-abstinent: M = 2.312, SE = 0.354).

In the 3 × 2 ANCOVA with mental health as the outcome measure, sexual activity level yielded a significant effect (F (1, 207) = 10.182, *p* < 0.01, η^2^_p_ = 0.047). Those who were sexually abstinent (M = 3.260, SE = 0.081) presented lower levels of mental health in comparison with non-abstinent inmates (M = 3.633, SE = 0.080). The effect due to partner status was non-significant.

Regarding associations with mental health, the Breuch–Pagan test yielded a significant result for heteroscedasticity (LM = 3.883, *p* < 0.05). Thus the HC3 heteroscedasticity-consistent standard error estimator was used [42] to run the regression model. The three-way interaction of sexual satisfaction × partner status × sexual activity level was not significant (Δ*R*^2^ = 0.001, F (2, 201) = 0.174, *p* > 0.05). By contrast, the two-way sexual satisfaction × sexual activity level interaction was statistically significant (Δ*R*^2^ = 0.016, F (2, 205) = 8.298, *p* < 0.01), whereas the sexual satisfaction × partner status interaction was not (Δ*R*^2^ = 0.007, F (1, 205) = 1.590, *p* > 0.05). In the former case, the conditional effects of sexual satisfaction at the values of the moderators showed lower levels of mental health only for those who were abstinent during the last six months. This result was found significant across the three levels of partner status (see Table 2 and Figure 1) and for the whole sample (sexual abstinent group: B = 0.176, SE = 0.089, *t* = 1.990, *p* < 0.05, 95% CI = (−0.346, 0.129)). No significant effect was observed for sexually active individuals.

As clearly can be seen in Figure 1, the interaction effect of sexual satisfaction × sexual activity presents a similar pattern for the groups of inmates without a partner, and in a current relationship outside or inside the prison. It is important to highlight that overall a decrease in sexual satisfaction of the sexually abstinent group is associated with a reduction of mental health levels, and the contrary, an increase in sexual satisfaction is related to an improvement in mental health.

## 4. Discussion

A direct relationship between sexual satisfaction and mental health was only found for the sexually abstinent group in this study. Partner status did not appear as a significant moderator. However, among those with a partner outside or inside prison, the sexually abstinent group showed lower levels of sexual satisfaction and mental health than sexually active inmates.

Again, sexual satisfaction was found to be significantly associated with mental health, as in other prison studies [36,50,51,78] and non-prison studies [52,53,54,56,57,58,59,60,63]. In this study, however, the sexual satisfaction, mental health association was only obtained for those who had remained sexually abstinent for at least the last six months. Previous research testing just one moderator has found that higher levels of sexual satisfaction were associated with higher levels of mental health only for prison inmates without a partner [50] and inmates who were sexually abstinent [51]. The current study examined the impact of both moderators, partner status and sexual activity level, together on the sexual satisfaction, mental health association. The results of this analysis showed that only the sexual satisfaction × sexual activity interaction was statistically significant. Neither the sexual satisfaction × partner status interaction nor the three-way interaction was significant. Thus a key implication of this study is that the lack of sexual relationships is more powerful as a moderator than the lack of a romantic relationship.

An important question here is why the lack of sexual relationships emerged in the regression analysis as significantly associated with mental health, whereas partner status did not. We speculate that sexual needs may be more important or basic than the emotional needs associated with romantic relationships. Sexuality, and more specifically sexual desire, comprises cognitive, emotional, and physiological processes and is consubstantial to the fact of being humans. Sexual desire may be a stimulus that sparks the inmates’ sensitivity to their lack of sexual satisfaction. Lack of sexual contact in prison has even been named by inmates as “sexual torture” [32]. By contrast, wishing to be in a romantic relationship in a prison where the pool of eligible partners may not be especially attractive, may produce either lower levels of reactance and/or lower levels of dissatisfaction with not having a partner. In addition, intimacy and emotional needs can be solved by other ties, like close friends [75]. This suggests that future research could profitably focus on the role of sexual desire levels as a means of dealing effectively with inmates’ sexual deprivation and/or the role that non-romantic, personal relationships contribute to improving their mental health. Hence, the damaging impact of being exposed to circumstances perceived as negative could be lessened by promoting positive interpersonal experiences and healthy interactions within inmates’ daily experiences.

Prison settings are an unconventional, yet potentially diagnostic, context in which to study the sexual satisfaction, mental health association. Similar contexts would be worth considering in other public health studies. The meaning of sexual satisfaction may be completely different for those who are sexually inactive or suffering from serious restrictions vis-a-vis sexual activities than for those who are sexually active. The current results have possible implications for other populations whose freedom to choose has been reduced or eliminated due to constraining situations or who are involved in more negative or difficult circumstances. As noted previously, strong associations between sexual satisfaction and mental health or other well-being related measures have been found in other populations afflicted by different medical conditions [70,71,72,73,74] or living in a more sexually restrained culture [77].

This study also found significantly lower levels of sexual satisfaction in the abstinent group. This result is consistent with previous research in prison [32,51]. Furthermore, there were parallel significant differences in sexual satisfaction between abstinent and sexually active inmates in both the groups with a partner outside and inside the prison (not all inmates with a partner were sexually active), but not for those without a partner. Having a partner and not having access to sexual relationships can generate even more reactance and/or create a worse position than not having a partner and sexual relationships. Additionally, sexually abstinent inmates showed low mental health. Presumably the abstinent inmates were in a worse situation and experiencing greater reactance to the loss of freedom with respect to their sexual lives than the sexually active inmates. All these results are consistent with previous research developed within prison contexts, highlighting inmates’ difficulties in meeting their sexual needs [32,33,34,35,51] and, as a consequence of this, presenting mental and emotional health problems [36].

Findings stemming from the two research questions of this study point to the crucial role sexual abstinence can have for mental health in some circumstances. Low sexual satisfaction (only for inmates with a partner outside or inside the prison) correlated with poorer mental health and a significant relationship between sexual satisfaction and mental health was observed in sexual abstainers. The abstinent group may be increasing their desire for sexual relationships due to their sexual deprivation [95]. Individuals wish to operate with a freedom to choose behaviors to satisfy their needs and if their freedom is reduced, threatened, or eliminated, individuals will become “motivationally aroused” to regain this freedom (see reactance theory [96,97]). Also, as seen in our results, this group is afflicted by sexual dissatisfaction, possibly the result of a large gap between their desires and their reality. Negative information and events (e.g., being abandoned by partners, losing friends, etc.) per se have been shown to have more impact on individuals’ judgments and well-being than positive ones (e.g., gaining friends, partners, etc.) (see “the bad is stronger than good approach” [98]), especially in stigmatizing contexts [99]. This association has also been found in romantic relationships in non-prison studies [69].

The reactance and the bad is stronger than good explanations complement one another but do differ. The reactance interpretation sees motivation as a triggering factor in the linkage between sexual abstinence and mental health. The “bad is better than good” interpretation places primary emphasis on evaluation per se as crucial in the sexual abstinence-mental health association. Future research might profitably examine whether the processes implied by one of these explanations is more applicable than the processes implied by the other and test this current study’s findings in other populations where individuals are afflicted by sexual deprivation or restriction due to different medical conditions or social factors.

In sum, this current investigation has found (a) lower levels of both sexual satisfaction and mental health in the sexually abstinent group, and (b) a stronger sexual satisfaction and mental health association in that group. Our perspective is that sexual satisfaction has been strongly correlated with mental health for the abstinent inmates likely because they are in a sexually worse or more deprived situation, and a similar, strong sexual satisfaction-mental health correlation should be observable in other comparably compromised situations or populations.

Our findings have important implications. First, inmates, especially those who are not sexually active, may benefit from prison policies that ease access to romantic and, especially, sexual relationships. We would note that inmates scoring higher on mental health have lower levels of misconduct [100] and lower recidivism rate after release [101]. Promoting positive mental health in prison inmates during incarceration and therefore increasing the likelihood of a successful reentry into society is a central concern with important consequences for public health, security, and the economy. According to this, clinical interventions to increase sexual access could be introduced to enhance inmates’ sexual satisfaction. This in turn should be associated with an increase in their mental health. Such changes, however, should take into account the risk profile of inmates because it may be an important variable influencing the choice of interventions.

Assuming inmates will not be able to engage in sexual activities with a partner, other policies and interventions may also be helpful. A shift in cognitions and/or attitudes might influence inmates’ evaluation of their sexual satisfaction. Cognitive restructuring techniques might be useful in this regard. Also, helping inmates to focus on other activities, especially ones that they pursue passionately, may relieve part of the distress associated with abstinence. In his dual theory of passion, Vallerand has shown that what he calls harmonious engagement in activities leads to psychological well-being [102]. Finally, increasing privacy in prison cells could facilitate masturbation as another way to obtain some sexual pleasure. Future research should address possible differences in sexual satisfaction between inmates who do, or do not, share their cells with other inmates. Also, it would be worthwhile to compare inmates living alone in a cell but in different prisons where inmates have more or less privacy (e.g., cameras in the rooms, prison officers entering in the cell without asking in advance, etc.).

We also believe that clinicians working with other populations who see their sexual freedom threatened (e.g., physical disabilities, older adults in nursing homes, etc.) can benefit from considering the implications of this study. Populations at risk of mental health problems should also be questioned about the presence or absence of sexual activity in their lives as a means of improving diagnosis and a more accurate intervention plan. Including sexual satisfaction in any diagnosis of mental health and its subsequent intervention seems sensible to consider, especially for those who have difficulties in meeting their sexual needs. Working on external impediments or barriers to having access to sexual relationships should be addressed too. Finally, clinical strategies aimed at reducing patients’ reactance and negative evaluations of their sexual deprivation coupled with helping patients discover and perform new highly motivating activities may help patients overcome part of the distress associated with their actual sexual situation.

Apropos of the limitations of this work, this study is correlational so causation is difficult to infer although we used a short-term longitudinal design. Also, a few participants affirmed engaging in homosexual behavior. Despite our stressing the confidentiality and anonymity of the study, homosexual contacts might have been underreported by the inmates. The Spanish context is conservative in character, where heteronormativity (the cultural assumption that heterosexuality is the only valid social norm) is tied deeply to culture [103]. These values are definitely prone to be found in prison inmates too [104]. In this context it is not easy to acknowledge engaging in homosexual behaviors. However, all the participants pointed out they felt very comfortable during the interview and disclosed information that they considered sensitive and important.

## 5. Conclusions

In sum, correctional systems often adopt deprivation as a solution to inmates’ sexual desires during incarceration. This study offers evidence regarding the importance of sexual satisfaction for their mental health, especially for abstinent inmates. A clear implication of this work is to urge prison administrators to find different solutions for inmates’ sexuality that helps them to deal with their sexual desires. But not only that, this study adds new evidence to highlight the importance of considering sexual satisfaction as a predictor of mental health especially in those populations whose freedom to engage in partnered sexual activity has been threatened. From a public health perspective, the association between sexual satisfaction and mental health can vary depending on an individual’s sexual activity level, as has been found in this study. Clinicians and health professional should take into consideration this possibility as part of their patients’ evaluation and intervention.

## Figures and Tables

**Table 1:** Correlations, means, and standard deviations of the variables considered in this study.

	Mean	SD	*%*_(1)_	1	2	3	4	5	6	7	8	9	10	11a	11b	11c	12	13	14
1. Sex (0 = male; 1 = female)			50.673		0.032	−0.033	−0.348 ***	0.102	−0.019	−0.259 ***	−0.070	−0.257 ***	−0.485 ***	−0.199 **	0.002	0.192 **	−0.364 ***	0.264 ***	0.048
2. Age	35.172	7.822				−0.108	0.156 *	0.006	−0.164 *	0.098	−0.027	−0.074	−0.146 *	0.006	0.074	−0.074	0.054	0.072	−0.014
3. Nationality (0 = Spanish; 1 = foreigner)			53.812				−0.269 ***	0.016	0.014	0.089	0.029	0.057	0.046	0.021	0.044	−0.061	0.120	−0.059	0.163 *
4. Total time in prison	54.337	48.623						−0.020	−0.010	0.178 **	0.165 *	0.139 *	0.265 ***	−0.162 *	−0.204 **	0.029	0.176 **	−0.125	−0.041
5. Time to parole	19.878	21.763							−0.088	0.029	0.025	0.001	−0.062	−0.081	0.082	0.004	−0.018	0.055	−0.141 *
6. Self-rated health	3.466	1.280								−0.165 **	−0.086	−0.148 *	0.053	−0.085	−0.106	0.180 **	−0.050	0.096	0.300 ***
7. Social loneliness	3.479	1.790									0.317 ***	0.274 ***	0.160 *	0.233 ***	−0.042	−0.189 **	0.265 ***	−0.211 **	−0.371 ***
8. Family loneliness	2.175	1.690										0.086	0.132 *	0.118	−0.098	−0.026	0.101	0.017	−0.090
9. Romantic loneliness	4.034	2.111											0.104	0.826 ***	−0.245 ***	−0.579 ***	0.547 ***	−0.563 ***	−0.198 **
10. Masturbation frequency	2.740	1.403												0.147 *	−0.181 **	0.022	0.143 *	−0.122	0.063
11a. No partner (0 = other; 1 = no partner)			34.081												−0.441 ***	−0.570 ***	0.600 ***	−0.449 ***	−0.164 *
11b. Partner inside (0 = other; 1 = partner inside)			38.565													−0.486 ***	−0.094	0.010	−0.024
11c. Partner outside (0 = other; 1 = partner outside)			27.354														−0.499 ***	0.428 ***	0.181 **
12. Sexual activity level (0 = active; 1 = abstinent)			45.291															−0.611 ***	−0.252 ***
13. Sexual satisfaction	2.483	3.449																	0.309 ***
14. Mental health	1.467	0.724																	

* *p* < 0.05; ** *p* < 0.01; *** *p* < 0.001. *%*_(1)_: Percentage of group with label “1” (females, foreigner, partner status, and abstinent) for dichotomous variables.

**Table 2:** Multiple regression analysis on mental health and conditional effects of sexual satisfaction at values of the moderators (partner status and sexual activity level).

	Mental Health			
	B	SE	*t*	95% CI
Sociodemographic and penitentiary variables				
Sex	−0.043	0.117	−0.366	(−0.273, 0.188)
Age	0.008	0.006	1.252	(−0.005, 0.021)
Nationality	0.359	0.092	3.899 ***	(0.178, 0.541)
Total time in prison	0.001	0.001	0.996	(−0.001, 0.003)
Time to parole	−0.004	0.002	−2.092 *	(−0.008, 0.001)
Personal, social, and sexual well-being variables				
Self-rated health	0.133	0.036	3.685 ***	(0.062, 0.204)
Social loneliness	−0.13	0.028	−4.592 ***	(−0.186, −0.074)
Family loneliness	−0.002	0.027	−0.072	(−0.056, 0.052)
Romantic loneliness	0.030	0.05	0.597	(−0.069, 0.129)
Masturbation frequency	0.058	0.038	1.515	(−0.017, 0.133)
Conditional effects				
Partner outside	−0.271	0.324	−0.835	(−0.910, 0.369)
Partner inside	−0.180	0.317	−0.566	(−0.805, 0.445)
Sexual activity level	−0.628	0.224	−2.800 **	(−1069, −0.186)
Sexual satisfaction	−0.108	0.121	−0.899	(−0.346, 0.129)
Two-way interaction model				
Sexual satisfaction × Partner status (outside)	0.221	0.124	1.781	(−0.024, 0.467)
Sexual satisfaction × Partner status (inside)	0.175	0.116	1.500	(−0.055, 0.404)
Sexual satisfaction × Sexual activity level	0.285	0.099	2.881 **	(0.090, 0.479)
*R* ^2^	0.355 ***			
Sexual satisfaction at values of the moderators				
Non-sexual abstinent—No partner	−0.108	0.121	−0.899	(−0.346, 0.129)
Non-sexual abstinent—Partner outside	0.113	0.063	1.791	(−0.011, 0.237)
Non-sexual abstinent—Partner inside	0.066	0.057	1.157	(−0.047, 0.179)
Sexual abstinent—No partner	0.176	0.089	1.990 *	(0.002, 0.351)
Sexual abstinent—Partner outside	0.398	0.104	3.835 ***	(0.193, 0.602)
Sexual abstinent—Partner inside	0.351	0.098	3.577 ***	(0.157, 0.544)

* *p* < 0.05; ** *p* < 0.01; *** *p* < 0.001. B, Unstandardized coefficient; SE, Standard Error.
